# Integrating multiple references for single-cell assignment

**DOI:** 10.1093/nar/gkab380

**Published:** 2021-05-25

**Authors:** Bin Duan, Shaoqi Chen, Xiaohan Chen, Chenyu Zhu, Chen Tang, Shuguang Wang, Yicheng Gao, Shaliu Fu, Qi Liu

**Affiliations:** Translational Medical Center for Stem Cell Therapy and Institute for Regenerative Medicine, Shanghai East Hospital, Bioinformatics Department, School of Life Sciences and Technology, Tongji University, Shanghai 200092, China; Translational Medical Center for Stem Cell Therapy and Institute for Regenerative Medicine, Shanghai East Hospital, Bioinformatics Department, School of Life Sciences and Technology, Tongji University, Shanghai 200092, China; Translational Medical Center for Stem Cell Therapy and Institute for Regenerative Medicine, Shanghai East Hospital, Bioinformatics Department, School of Life Sciences and Technology, Tongji University, Shanghai 200092, China; Translational Medical Center for Stem Cell Therapy and Institute for Regenerative Medicine, Shanghai East Hospital, Bioinformatics Department, School of Life Sciences and Technology, Tongji University, Shanghai 200092, China; Translational Medical Center for Stem Cell Therapy and Institute for Regenerative Medicine, Shanghai East Hospital, Bioinformatics Department, School of Life Sciences and Technology, Tongji University, Shanghai 200092, China; Translational Medical Center for Stem Cell Therapy and Institute for Regenerative Medicine, Shanghai East Hospital, Bioinformatics Department, School of Life Sciences and Technology, Tongji University, Shanghai 200092, China; Translational Medical Center for Stem Cell Therapy and Institute for Regenerative Medicine, Shanghai East Hospital, Bioinformatics Department, School of Life Sciences and Technology, Tongji University, Shanghai 200092, China; Translational Medical Center for Stem Cell Therapy and Institute for Regenerative Medicine, Shanghai East Hospital, Bioinformatics Department, School of Life Sciences and Technology, Tongji University, Shanghai 200092, China; Translational Medical Center for Stem Cell Therapy and Institute for Regenerative Medicine, Shanghai East Hospital, Bioinformatics Department, School of Life Sciences and Technology, Tongji University, Shanghai 200092, China

## Abstract

Efficient single-cell assignment is essential for single-cell sequencing data analysis. With the explosive growth of single-cell sequencing data, multiple single-cell sequencing data sources are available for the same kind of tissue, which can be integrated to further improve single-cell assignment; however, an efficient integration strategy is still lacking due to the great challenges of data heterogeneity existing in multiple references. To this end, we present mtSC, a flexible single-cell assignment framework that integrates multiple references based on multitask deep metric learning designed specifically for cell type identification within tissues with multiple single-cell sequencing data as references. We evaluated mtSC on a comprehensive set of publicly available benchmark datasets and demonstrated its state-of-the-art effectiveness for integrative single-cell assignment with multiple references.

## INTRODUCTION

Single-cell transcriptomics is indispensable for identifying and characterizing the cellular composition of complex tissues and organisms (1–6). In the process of single-cell RNA sequencing data analysis, cell type identification is the fundamental step for downstream analysis. Recently, cell type assignment strategies without prior marker gene annotations have been presented (7–13). These strategies select one labeled single-cell sequence dataset as the reference to automatically assign query cells with cell types in the reference by measuring the transcriptional profile similarity between the query cell and reference cells, and prior cell type-specific marker gene information is not necessarily required in this process. The single-cell assignment strategy has been indicated to be helpful for cell type identification, but it relies heavily on the quality and quantity of the reference datasets (14). Most existing methods, however, are designed to apply one preselected reference dataset for the assignment, leading to two challenges; i.e. (i) the limited number of cell types and cells in one reference will substantially influence the assignment results, which may result in unassigned or incorrect cell assignments and (ii) the reliability of the reference dataset will also influence the assignment results. Noise or incorrectly annotated cell types in the reference dataset will result in incorrect cell assignments (14). These issues are expected to be addressed by integrating multiple single-cell reference datasets for the same kind of tissue (7,11,12), and an efficient integration strategy is required.

Basically, there are three levels of integration strategies: the data level, the algorithm level and the decision level. Data-level integration involves the integration of multiple datasets into one dataset by reducing batch effects. Algorithm-level integration involves the design of efficient algorithms for model integration, while the different datasets remain separate. For example, multitask learning can be considered an algorithm-level integration strategy (15). Decision-level integration treats the data and models separately while integrating individual assignment results by an ensemble strategy. Currently, only a few methods have been specifically presented for single-cell assignment with multiple references (7,11,12). These methods mainly adopt two strategies: (i) data-level integration by integrating multiple datasets into one reference for single-cell assignment (12) and (ii) decision-level integration by performing separate single-cell assignments for individual reference datasets, where the final cell type identification is achieved by ensembling the individual assignment results (7,11). Although both strategies take advantage of multiple reference datasets, they have limitations: (i) the current data-level integration method relies heavily on batch-effect correction methods. These batch-effect correction methods commonly face the problem of overcorrection and often transform the original feature (gene expression) into a comprehensive variable (12,16–20), which will adversely affect the assignment performance. (ii) The current decision-level integration method does not consider the relationships among the reference datasets during training; therefore, it does not obtain the maximum values of these references.

In this study, we propose mtSC, which is an efficient integration strategy for single-cell assignment with multiple references that takes advantage of both algorithm-level and decision-level integration. We do not adopt data-level integration to avoid the overcorrection of the batch effect. In this way, mtSC provides a flexible and dynamic way to include related reference datasets of interest. In our previous study (13), metric learning was proven to be effective for single-cell assignment. Specifically, mtSC presents a multitask deep metric learning-based framework for single-cell assignment with multiple references. It regards each dataset as an individual task to train a multitask-based deep neural network for single-cell assignment rather than directly integrating all datasets into one reference (data-level integration) or directly combining the individual assignment results (decision-level integration). Intuitively, multitask learning for multiple reference datasets can underline the common information existing in multiple references for batch-effect correction. The different information available in multiple references can be complementary to improve the overall assignment performance. As a result, we evaluated mtSC on 27 benchmark datasets for different tissues and proved its state-of-the-art effectiveness for integrative single-cell assignment with multiple references.

## MATERIALS AND METHODS

### Multiple reference datasets for benchmarking integrative single-cell assignment

We evaluated mtSC on 27 single-cell assignment benchmark datasets which were curated from four studies including three tissues: peripheral blood mononuclear cells (PBMCs) (21,22), the brain (23,24) and the pancreas (25–28) (Supplementary Table S1). For all 27 datasets, cell types with <10 cells were removed because they do not contain enough information and are unreliable for subsequent assignment. In the dataset generated by Segerstolpe *et al.* (27), cells labeled ‘not applicable’ were removed. In the dataset generated from Xin *et al.* (28), cells labeled ‘alpha.contaminated’, ‘beta.contaminated’, ‘gamma.contaminated’ and ‘delta.contaminated’ were removed because they likely corresponded to cells of lower quality. In the dataset generated from Tasic *et al.* (24), four cell types, ‘L6b’, ‘Pvalb’, ‘Sst’ and ‘Vip’, were retained to match the names of cell types in the other three brain datasets (23). For ‘PBMC-Mereu’ (22), 12 datasets were used (Supplementary Table S1), excluding the Smart-seq2-based dataset, which was too small.

### Data preprocessing

The data preprocessing step of mtSC consists of three parts: cell quality control, rare cell type filtering and gene expression profile formatting. mtSC evaluates the cell quality based on strict criteria following three commonly used considerations, namely, the number of genes detected (default >500), the number of unique molecular identifiers induced (default >1500) and the percentage of mitochondrial genes detected (default <10% among all genes). Only cells satisfying all three criteria are retained to construct the reference data. Then, all the datasets were normalized, i.e. scaling to 10 000 and then with log(counts+1). Next, mtSC removes rare cell types with <10 cells because such cell types do not contain enough information and are unreliable for subsequent assignment. Finally, all the datasets are processed into an identical format, i.e. expression profiles with the union of the genes in all the multiple reference datasets. If the query dataset does not contain a gene in the gene union of the reference datasets, the column of the gene will be filled with zeros.

### Model learning of mtSC for integrative single-cell assignment

In the model learning stage, mtSC establishes a multitask deep metric learning (DML) model trained on multiple reference datasets simultaneously. For DML, the N-pair loss (29) is used as the loss function. The DML neural network that we used contains an input layer, a hidden layer and an output layer. The input layer has a number of nodes equal to the genes of the reference. The hidden layer and output layer have 500 and 20 nodes, respectively.

mtSC extends single-task DML to a multitask learning framework by sharing the model parameters among tasks. Given all or part of *m* related learning tasks, multitask learning aims to improve the model learning for a single task by utilizing the knowledge contained among the *m* tasks (30). On the basis of the single-task DML, we add the loss of all the tasks together and update the parameters through the backpropagation algorithm in each iteration. All the tasks share model parameters of all the layers during the training process.

The application of the *N*-pair loss consists of two parts: batch construction and calculation. For the batch construction of the *N*-pair loss, $\{ {( {{x_1},\;x_1^ + } )\cdot{\rm{\;}}\cdot{\rm{\;}}\cdot,{\rm{\;}}( {{x_N},x_N^ + } )} \}$ is defined as *N* pairs of cells from *N* different cell types, in which *x_i_*≠*x_j_* ∀ *i*≠*j*. Then, *N* tuples denoted by $\{ {{S_i}} \}_{i\; = {\rm{\;}}1}^N$ are built from the *N* pairs, where ${S_i}{\rm{\;}} = {\rm{\;}}\{ {{x_i},{\rm{\;}}x_1^ + ,{\rm{\;}}x_2^ + ,{\rm{\;}}\cdot{\rm{\;}}\cdot{\rm{\;}}\cdot,{\rm{\;}}x_N^ + } \}$. Here, ${x_i}$ is the query for ${S_i}$, $x_i^ +$ is a positive example, and $x_j^ + ( {{\rm{}}j{\rm{\;}} \ne {\rm{\;}}i{\rm{}}} )$ are the negative examples. ${x_i}$ and $x_i^ +$ are two cells of the same cell type, and $x_j^ +$ are the cells with different cell types different from ${x_i}$.

The calculation of the *N*-pair loss can be formulated as follows:

$$\begin{eqnarray*}&&{L_{N - {\rm{pair}}}}(\{ \left( {{x_i},{\rm{\;}}x_i^ + } \right)\} _{i\; = {\rm{\;}}1}^N;f){\rm{\;}} \\ &&\quad = {\rm{\;}}\frac{1}{N}\mathop \sum \limits_{i\; = {\rm{\;}}1}^N {\rm{log}}(1 + \mathop \sum \limits_{j \ne i} {\rm{exp}}\left( {f_i^{\rm{T}}f_j^ + - f_i^{\rm{T}}f_i^ + } \right)) \end{eqnarray*}$$

in which *f*(·; *θ*) is an embedding kernel defined by a deep neural network, ${f_i}$ and $f_i^ +$ are embedding vectors of two cells of the same cell type and $f_j^ +$ are embedding vectors of cells whose cell types are different from ${x_i}$.

### Model parameters of mtSC

The neural network model was implemented in Python with PyTorch. The Adam optimizer is used as the optimizer. The initial learning rate was set to 0.0005, and the other parameters of the Adam optimizer were the defaults. The number of training epochs was set to 300. The L2 regularization rate was set to 0.05. Additionally, sensitivity analysis was performed for these parameters (Supplementary Figure S1 and Supplementary Table S2). The results showed that mtSC is not sensitive to these parameters with a good robustness.

### Query cells assignment

After model learning, the trained parameter-shared deep metric learning network (PS-DMLN) was obtained. Then, query cell assignment can be performed. First, cell quality control is optional for users, and the query data were scaled to 10 000 and normalized with log(counts+1). If the query dataset does not contain a gene in the gene union of the reference datasets, the column of the gene will be filled with zeros. Next, with the trained model, the query cells were transformed to the same embedding space as the transformed references. Then, the transformed query dataset was assigned to each transformed reference dataset. Specifically, for each transformed reference dataset, mtSC carried out a cell search by measuring the transcriptional similarity between query cells and the cell cluster centroid of each transformed reference dataset. In our study, the Pearson correlation coefficient was adopted as in our previous study (13). Finally, the query cells obtained the predicted cell types with the highest similarity among all the transformed reference datasets.

### Benchmarking existing tools for integrative single-cell assignment

To evaluate the performance of mtSC, three existing tools for multiple references were compared: scmap-cluster (11), SingleR (7) and Seurat v3 (12). In all the analysis of our study, for a fair comparison, ‘threshold = 0’ was set for scmap-cluster because mtSC does not assign query cells with an ‘unassigned’ result. For SingleR, ‘fine.tune = FALSE’ was set because the fine-tuning process of SingleR is extremely time consuming (14). For Seurat v3, all the parameters were the default values. For all benchmarks, scmap-cluster, SingleR and Seurat v3 were trained and tested with CPU Intel Xeon Platinum 8165 2.3-3.7GHz. As a deep learning based model, mtSC was trained with GPU 1080Ti and tested with the same CPU as those of the other methods.

To further prove the superiority of mtSC, we compared mtSC with a broader selection of other comparable single reference based methods listed in previous benchmark (14), including CHETAH (31), scID (32), ACTINN (10), SVM (33), NMC (33), RF (33), LDA (33) and kNN(*k* = 9) (33), in which CHETAH, scID, ACTINN and NMC cannot support decision level integration and LDA cannot support data level integration. For CHETAH, ‘thresh = 0’ was set. For other methods, all the parameters were the default values.

### Evaluation criteria for integrative single-cell assignment

The macro-F1 score was used to evaluate the performance of the different methods. First, the precision and recall of each cell type were calculated. Then, the macro-F1 score was calculated as listed below:

$$\begin{equation*}{\rm{macro}} - {\rm{F}}1{\rm{\;}} = \frac{1}{N}\;\mathop \sum \limits_{i\; = \;1}^N \frac{{2*Precisio{n_i}*Recal{l_i}}}{{Precisio{n_i} + Recal{l_i}}}\end{equation*}$$

in which *N* denotes the number of cell types in a dataset and $Precisio{n_i}$ and $Recal{l_i}$ are the precision and recall of the *i*-th cell type in the dataset.

### Multiple reference databases built into mtSC

To facilitate the broad application of mtSC for single-cell assignment with multiple reference datasets, we provide not only the mtSC Python package but also the pretrained models for the 27 datasets tested in our study for direct utilization (Supplementary Table S1).

These multiple reference datasets covered three tissues: the brain, pancreas and PBMCs. They can be directly and successfully applied to the related single-cell assignment task. The Python package and the pretrained assignment models can be downloaded from GitHub (https://github.com/bm2-lab/mtSC).

## RESULTS

### Overview of mtSC

**Figure 1.**
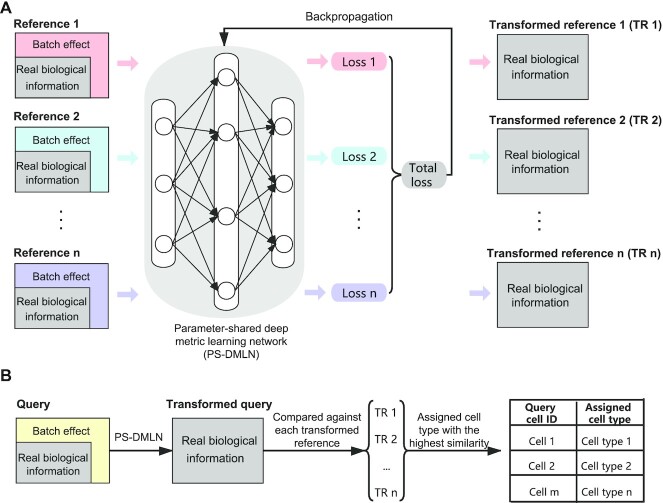
The mtSC workflow. (**A**) The model learning process of mtSC. Each dataset is considered a single task, and a corresponding loss is calculated. All the losses are added together and utilized to update the model parameters through a backpropagation algorithm. (**B**) The cell assignment process of mtSC. The trained PS-DMLN is utilized to transform the query cells. Then, the transformed query cells are compared against transformed reference cells, and the predicted cell type with the highest similarity among all the transformed reference datasets is obtained.

mtSC is a multitask deep metric learning-based framework for single-cell assignment with multiple reference datasets. Generally, the information contained in each reference dataset can be divided into two parts: batch-effect noise and underlying real biological information (Figure 1). We assumed that the common information of each reference dataset is the underlying real biological information, since although batch effects generated from different experiments exist, the underlying real biological information should remain the same across the different datasets generated from the same tissues. By applying multitask deep metric learning, mtSC is able to obtain the underlying real biological information of each reference dataset. Specifically, mtSC comprises two main steps: model learning and cell assignment (Figure 1 and see Materials and Methods section).

**Figure 2.**
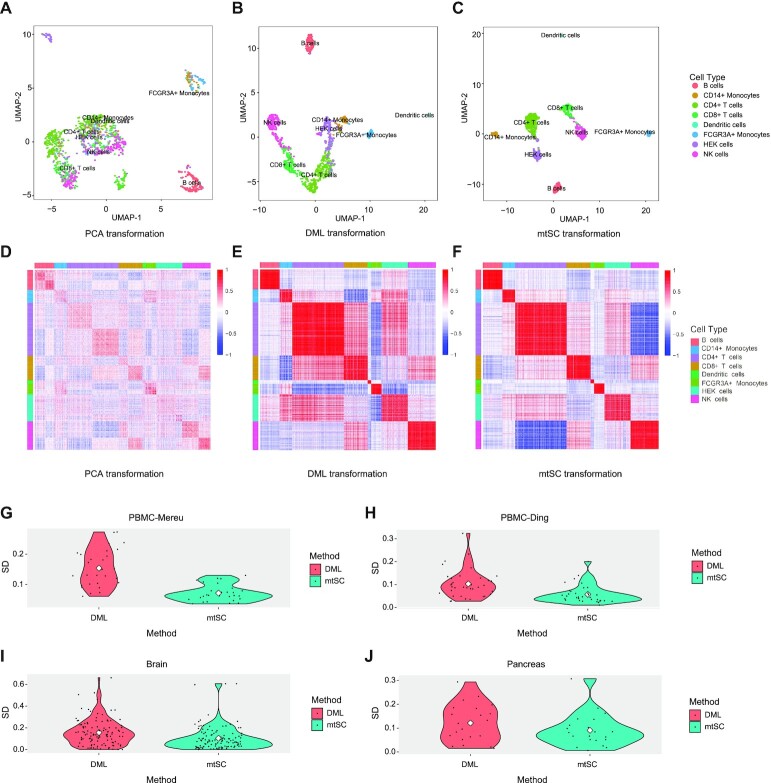
Comparison of the transformations of PCA, DML and mtSC. (**A**) Visualization of the clustering results with UMAP after PCA transformation on the ‘Pbmc_chromium2’ dataset. (**B**) Visualization of clustering results with UMAP after DML transformation on the ‘Pbmc_chromium2’ dataset. (**C**) Visualization of the clustering results with UMAP after mtSC transformation on the Pbmc_chromium2 dataset. (**D**) Similarity heatmap calculated with the Pearson correlation coefficient after PCA transformation on the ‘Pbmc_chromium2’ dataset. (**E**) Similarity heatmap calculated with the Pearson correlation coefficient after DML transformation on the ‘Pbmc_chromium2’ dataset. (**F**) Similarity heatmap calculated with the Pearson correlation coefficient after mtSC transformation on the ‘Pbmc_chromium2’ dataset. (**G**) The comparison of the SD of similarity between any two cell types within ‘PBMC-Mereu’ transformed by DML and mtSC. The white diamond represents the mean value. SD stands for standard deviation. (**H**) The comparison of the SD of similarity between any two cell types within ‘PBMC-Ding’ transformed by DML and mtSC. (**I**) The comparison of SD of similarity between any two cell types within the brain transformed by DML and mtSC. (**J**) The comparison of SD of similarity between any two cell types within the pancreas transformed by DML and mtSC.

In the model learning stage (Figure 1A), mtSC establishes a multitask deep metric learning model trained on multiple reference datasets simultaneously. First, deep metric learning (DML) is applied to learn an optimal measurement fitting the relationship among cells in individual datasets, and the N-pair loss (29) is used as the loss function for model training (Figure 1A and see Materials and Methods section). By applying DML, an optimal measurement is learned based on the prior sample similarity and dissimilarity information, making cells with the same label more similar and cells with different labels more dissimilar (Figure 2A, B, D and E and Supplementary Figure S2). Then, mtSC extends DML to a multitask learning framework by sharing model parameters between tasks to construct a parameter-shared deep metric learning network (PS-DMLN) (Figure 1A and see Materials and Methods section), which is able to utilize the complementary information from different reference datasets to boost the cell assignment performance. Finally, mtSC obtains individual transformed references under the multitask learning framework, where the real biological information is uncovered and the batch effect between different reference datasets is reduced. These transformed references will be used in the following cell assignment process.

In the cell assignment stage (Figure 1 and see Materials and Methods section), by applying the model trained in the previous stage, the query dataset is first transformed to the same embedding space as that of the transformed reference datasets. Then, the transformed query dataset is assigned to proper cell types by comparing their transcriptional profiles with individual transformed reference datasets. Specifically, for each transformed reference dataset, mtSC carries out a cell search by measuring the transcriptional similarity between the transformed query cells and the cell cluster centroid of each transformed reference dataset. Finally, the query cells are assigned to the proper cell type with the highest similarity among all the transformed reference datasets, which is a kind of decision-level integration strategy based on the individual assignment results.

### Validation of the rationale of mtSC

We first present an intuitive validation of the rationale of mtSC for single-cell assignment with multiple references. To this end, we collected 27 datasets (Supplementary Table S1) from four studies of three tissues: two studies on peripheral blood mononuclear cells (PBMCs) (21,22), one study on the brain and one study on the pancreas. For PBMCs, the first study (22) involved 12 datasets from 12 different sequencing platforms (‘PBMC-Mereu’), and the second study (21) involved 7 datasets from 7 different sequencing platforms (‘PBMC-Ding’). For brain tissue, the study involved four brain datasets (23,24) with different sources. For pancreas tissue, the study involved four commonly used pancreas datasets (25–28).

For illustration purposes, we first used ‘PBMC-Mereu’ as an example to demonstrate the rationale of mtSC. The multiple datasets contained in ‘PBMC-Mereu’ were used as multiple references to train the model, and then the transformed reference datasets were obtained. To intuitively show why and how mtSC works, we compared the clustering results of the reference datasets transformed after principal component analysis (PCA), deep metric learning (DML) and multitask deep metric learning (mtSC) by uniform manifold approximation and projection (UMAP) respectively (Figure 2A–F and Supplementary Figure S2). For illustration purposes, we used the ‘Pbmc_chromium2’ dataset in ‘PBMC-Mereu’ as an example plot (Figure 2A–F and Supplementary Table S1). As we can see in Figure 2A and B, compared to PCA, DML is able to make cells within the same cell type become more similar, while cells among different cell types become more dissimilar, as shown in our previous work on scLearn (13). However, several specific cell types, such as CD4+ T cells and CD8+ T cells, cannot be separated properly by DML due to inherent noise. Nevertheless, with the help of other datasets in ‘PBMC-Mereu’, mtSC, which is a multitask learning-based DML framework, was able to further distinguish CD4+ T cells and CD8+ T cells properly (Figure 2D–F).

To further show the generalizability of mtSC, all four studies were used in the following tests. Intuitively, we assume that if the reference datasets transformed by multitask learning indeed obtained the common information among all the datasets, the similarity between any two cell types within each reference dataset transformed by mtSC should be more consistent among all the reference datasets than those transformed by DML. The comparison results are shown in Figure 2G and H. As expected, mtSC obtained more consistent results than DML in all four studies, further validating the superiority and generalizability of mtSC. More detailed information on these comparisons is shown in Supplementary Figure S3 and Supplementary Tables S3–S6.

### Benchmarking mtSC with available integrative single-cell assignment strategies for multiple references

Current methods for single-cell assignment with multiple references mainly adopt two strategies: (i) Data-level integration first integrates multiple reference datasets into one reference dataset by reducing the batch effects (12), and then single-cell assignment can be performed on this integrated reference by any reported method. (ii) Decision-level integration performs single-cell assignment for individual reference datasets separately, and the final cell type identification is achieved by ensembling individual assignment results (7,11). To further prove the superiority of mtSC, mtSC was compared against traditional data-level and decision-level integration strategies.

**Figure 3.**
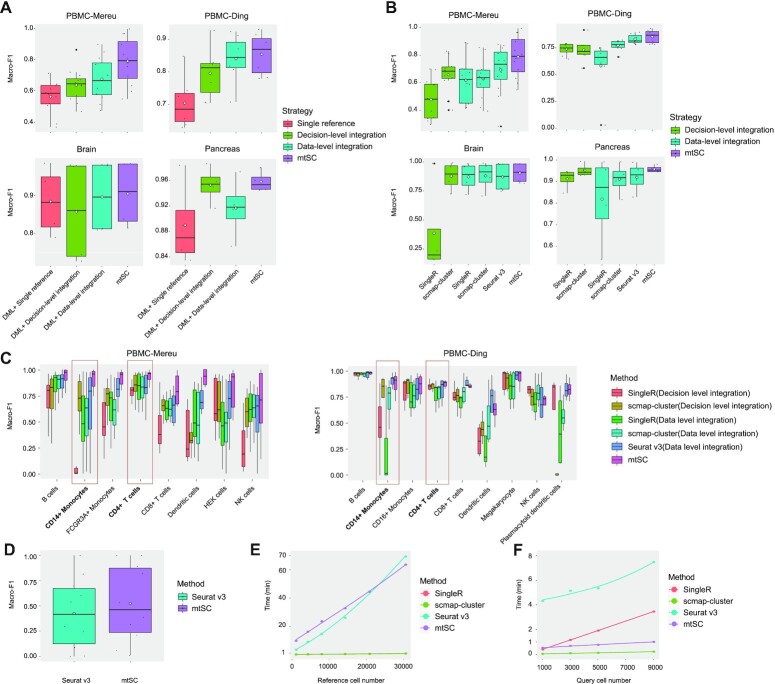
Evaluating the performance of mtSC with different multiple references based single-cell assignment strategies and methods. (**A**) The macro-F1 scores of the different single-cell assignment strategies for ‘PBMC-Mereu’, ‘PBMC-Ding’, ‘Brain’ and ‘Pancreas’, respectively. The white diamond represents the mean value. (**B**) The macro-F1 scores of the different existing tools with different integration strategies for ‘PBMC-Mereu’, ‘PBMC-Ding’, ‘Brain’ and ‘Pancreas’, respectively. (**C**) The Macro-F1 of each cell type for different methods on ‘PBMC-Mereu’ and ‘PBMC-Ding’ studies. The red frames and bold text are cell types with greater improvement on both studies. (**D**) The macro-F1 of single cell assignment for Seurat v3 and mtSC, where specific cell types only exist in one of the multiple references for ‘Brain’ datasets. (**E**) Single CPU (SingleR, scmap-cluster and Seurat v3) and single GPU (mtSC) execution time to train a model based on the references for existing tools. Solid lines are loess regression fitting (span = 2), implemented with R function geom_smooth(). (**F**) Single CPU (SingleR, scmap-cluster, Seurat v3 and mtSC) execution time to test query cells for existing tools. Solid lines are loess regression fitting (span = 2), implemented with R function geom_smooth().

In this test, ‘PBMC-Mereu’, ‘PBMC-Ding’, ‘Brain’ and ‘Pancreas’ were used as the benchmark studies to evaluate the performance of different integration strategies for single-cell assignment with multiple reference datasets (Figure 3A and Supplementary Table S7). For data-level integration, each dataset among the multiple datasets was used as the query, and the others were integrated into one dataset as the reference. Seurat v3 (12) was used to integrate the multiple reference datasets. Then, DML was used to train the integrated reference dataset and predict the cell types (DML+data-level integration). For the decision-level integration strategy, each dataset among the multiple datasets was used as the query, and the other datasets were taken as the multiple reference datasets. Then, DML was performed to predict cell types with the highest similarity of cell types among all the reference datasets (DML+decision-level integration). In addition, for both integration strategies, we also used only one reference for single-cell assignment as the baseline, and DML was also performed for consistent comparisons. Specifically, in this case, each of the multiple datasets was used as a query dataset, and each of the remaining datasets was taken as an individual reference. Then, DML was used to train the model to predict the cell types. For each query dataset, the average macro-F1 score for all the remaining individual references was calculated as its final result (DML+single reference). It should be noted that in our study, the integration of multiple reference datasets can help to address the issue that the specific cell types of query datasets don’t exist in one reference when multiple references are available, therefore, for a fairness comparison purpose, the benchmark scenario is set as all the query cell types are included in the reference datasets, and the macro-F1 score was used as the evaluation metric.

In Figure 3A and Supplementary Table S7, it can be clearly seen that for all four strategies, the multiple reference-based integration strategies generally achieved a better performance than the single reference-based strategies, while among all the multiple reference-based integration strategies, mtSC obtained the best performance, proving its superior generalizability for single-cell assignment with multiple references.

### Benchmarking mtSC with existing tools for single-cell assignment with multiple references

Although mtSC has been proven to be more effective than other traditional integration strategies, it was further compared to existing tools for single-cell assignment with multiple references. These tools include scmap-cluster (11), SingleR (7) and Seurat v3 (12). scmap-cluster and SingleR adopt a decision-level integration strategy, and Seurat v3 adopts a data-level integration strategy for single-cell assignment with multiple references. Since, theoretically, scmap-cluster and SingleR can also adopt the data-level integration strategy, for a comprehensive comparison, in the evaluation of the data-level integration strategy, Seurat v3, scmap-cluster and SingleR were all tested.

Specifically, each dataset in each study (‘PBMC-Mereu’, ‘PBMC-Ding’, ‘Brain’ and ‘Pancreas’) was considered a query dataset, and the other datasets were taken as the multiple reference datasets. In the evaluation of decision-level integration, scmap-cluster and SingleR were tested with their own designed processes for multiple reference assignment; i.e. the model was trained on each reference, and the cell type was identified with the highest similarity among all the multiple references. In the evaluation of the data-level integration strategy, the reference datasets were first integrated into one integrative dataset with Seurat v3; then, Seurat v3, scmap-cluster and SingleR were all implemented on this integrative reference for single-cell assignment.

From Figure 3B and Supplementary Table S8, it is clear that the data-level integration-based tools generally performed better than the decision-level integration-based tools, while mtSC achieved the best performance for all four studies, further proving its superiority. Additionally, a broader comparison between mtSC and other comparable single reference based methods (10,14,31–33) was performed and further proved the superiority of mtSC (Supplementary Figure S4 and Supplementary Table S9 and see Materials and Methods section). To further investigate in which cell types mtSC achieved the greatest improvement upon other competing methods, we compared the macro-F1 for each cell type on ‘PBMC-Mereu’ and ‘PBMC-Ding’ studies, since the two studies shared several of the common cell types. As shown in Figure 3C and Supplementary Table S10, in the common cell types of the two studies, mtSC obtained greater improvement for CD14+ Monocytes and CD4+ T cells. CD14+ Monocytes are very similar to CD16+ Monocytes and FCGR3A+ Monocytes and CD4+ T cells are very similar to CD8+ T cells, so they are difficult to be distinguished in the original feature space (see Figure 2A–F and Supplementary Figure S2). With multitask deep metric learning, mtSC can integrate the shared information of each dataset to facilitate the correct assignment of similar cell types. This result is consistent with our prior validation of the rationale of mtSC. It should be noted that data-level integration method, such as Seurat v3, rather than decision-level integration, is limited with overcorrection issue, to further prove the superiority of mtSC in terms of overcorrection, an additional comparison between Seurat v3 and mtSC is performed. In this scenario, tissues from individual controls might have rare cell types not captured in other experiments using the same tissue, and overcorrection may occur when integrating these datasets together. Therefore, we evaluated the assignment performance of mtSC and Seurat v3 with rare cell types that only appeared in one of the multiple references. Specifically, in the four ‘Brain’ datasets, there are five cell types which only appear in two datasets, each of which can be considered as query dataset, and the other dataset can be combined with the rest two datasets together as multiple references. Then the macro-F1 scores of the five cell types are calculated with Seurat v3 and mtSC. The result is shown in Figure 3D and Supplementary Table S11. We can see that mtSC obtains better performance than Seurat v3, proving its superiority in terms of overcorrection.

The computational efficiency of single-cell assignment methods is important as cell number increases. The execution time for single cell assignment consists of training time and querying time, and saving the querying time is more important than the training time, since users can use the pre-trained models to query cells. Our comparison of execution time (Figure 3E and F and Supplementary Tables S12 and S13 and see Materials and Methods section) indicated that mtSC consumes less time than that of Seurat v3 to train a model. For querying process, mtSC is also very fast (<1 min for 9000 query cells).

### mtSC performs increasingly better as the number of reference datasets increases

In this study, we further investigated the impact of the number of references on the performance of mtSC. We used ‘PBMC-Ding’ as an example. mtSC was trained with different numbers of reference datasets, and then the corresponding macro-F1 score was calculated to show the trend in the performance as the number of reference datasets increased. Specifically, each time, we randomly selected one dataset from the 7 datasets as the query dataset and selected 2 to 6 datasets without replacement from the remaining datasets as the multiple reference datasets (see Materials and Methods section). This process was repeated five times to reduce randomness.

**Figure 4.**
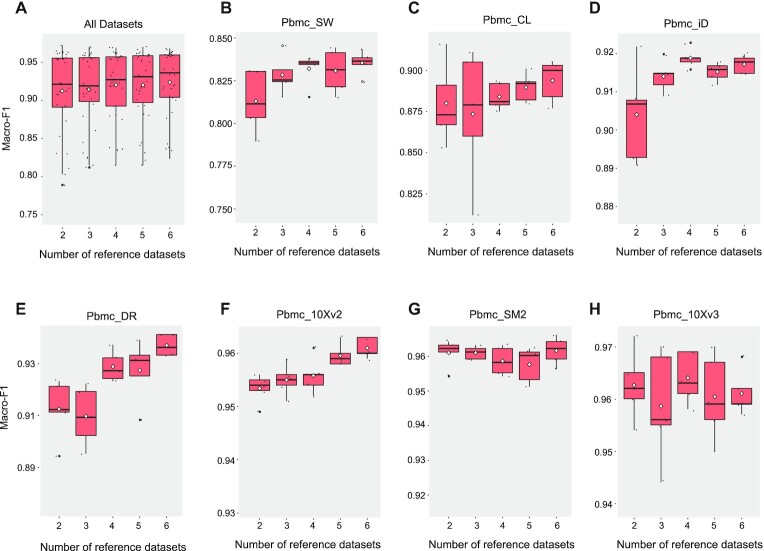
The performance of mtSC as the number of reference datasets increases for ‘PBMC-Ding’. (**A**) The Macro-F1 scores for ‘PBMC-Ding’. The white diamond represents the mean value. (**B**) The Macro-F1 scores for query dataset ‘Pbmc-SW’. (**C**) The Macro-F1 scores for query dataset ‘Pbmc-CL’. (**D**) The Macro-F1 scores for query dataset ‘Pbmc-iD’. (**E**) The Macro-F1 scores for query dataset ‘Pbmc-DR’. (**F**) The Macro-F1 scores for query dataset ‘Pbmc-10Xv2’. (**G**) The Macro-F1 scores for query dataset ‘Pbmc-SM2’. (**H**) The Macro-F1 scores for query dataset ‘Pbmc-10Xv3’.

As shown in Figure 4 and Supplementary Table S14, mtSC generally performs increasingly better as the number of reference datasets increases (Figure 4A). More detailed information on each query dataset is shown in Figure 4B–H. It is clearly seen that when the performance is relatively lower in terms of the macro-F1 score in the beginning, the improvement becomes more evident as the number of reference datasets increases (Figure 4B). Such an improvement obtained by increasing the related references is of great importance in the era of explosive growth of single-cell datasets. To this end, mtSC can be applied to integrate a growing number of single-cell datasets to obtain a better performance for single-cell assignment.

### mtSC enables cross-species single-cell assignment

Single-cell assignment depends heavily on the reference sequencing datasets. For specific tissues, sequencing datasets are difficult to collect and rarely available. For example, due to ethical issues, single-cell sequencing data for the human brain are rarely available, and it is of great importance to be able to take advantage of tissue sequencing data from other model animals, such as monkeys or mice, for human brain cell annotations. To this end, we investigated the potential utility of mtSC in integrating mouse brain single-cell sequencing data for improved human brain cell assignment. In this way, one human brain dataset (23) and four mouse brain datasets (23,24) were collected. We first randomly selected a small portion of the human brain dataset, together with the other four mouse brain datasets, as multiple references to mimic the scenario where human brain single-cell sequencing data are rarely available, while mouse brain single-cell sequencing data are relatively easy to collect. Then, the single-cell assignment is performed by using the remaining human brain dataset as the query. In this test, DML was used as the baseline. It should be noted that for a fair comparison, decision-level integration is not adopted by mtSC in this test. Only the reference human brain dataset was used to assign the remaining human brain cells, while other mouse brain datasets were included in the model training under only the multitask learning framework and were not considered at the final decision level. The rationale behind this test is that due to the heterogeneity between different species, the mouse brain datasets can have advantages during model training, while they should be avoided when applying directly at the final decision level for human brain assignment.

**Figure 5.**
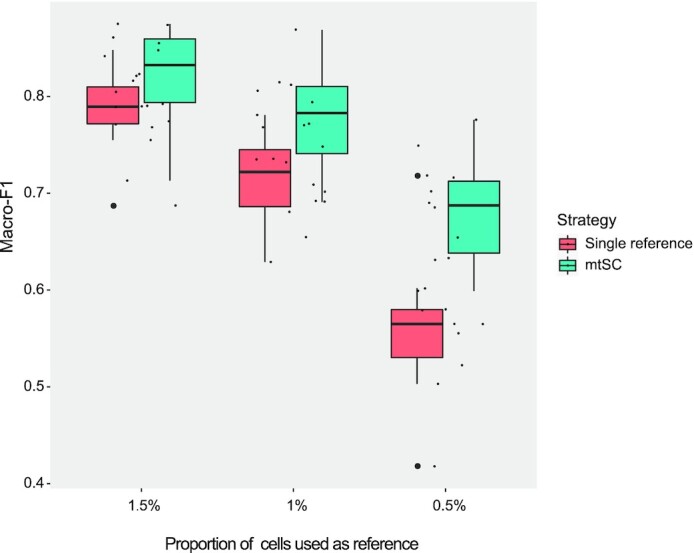
The performance of mtSC for cross-species single-cell assignment. mtSC trained with a mixture of multiple references outperformed the methods trained with only the human brain dataset as the reference. The smaller the proportion of the human brain dataset taken as the reference, the better the improvement in mtSC is.

From Figure 5 and Supplementary Table S15, it is clear that mtSC trained with a mixture of multiple references outperformed the methods trained using only the human brain dataset as the reference. In addition, such improvement becomes more evident when the proportion of human brain reference datasets decreases, further indicating the effectiveness of mtSC for cross-species single-cell assignment when there are rare reference tissue sequencing data available.

## DISCUSSION

In this study, we present a novel, flexible and generalized multitask deep metric learning-based framework, mtSC, for single-cell assignment based on multiple references. With the development of single-cell sequencing technologies, substantial single-cell reference datasets are accumulated, which can be integrated to improve cell annotations. Previous strategies for single-cell assignment with multiple references rely on data- or decision-level integration, while limitations remain. Different from the previous strategies, mtSC regards each reference dataset as a task, and different tasks can be complementary to improve single-cell assignment, while the overcorrection of the batch effect can be avoided. Such a novel integration strategy provides a flexible and reliable way to integrate related reference datasets. Our comprehensive validation and benchmark on 27 previously published datasets indicated that mtSC has achieved state-of-the-art performance for single-cell assignment with multiple references.

Two additional advantages of mtSC were proven in this study: (i) mtSC performs increasingly better as the number of reference datasets increases and (ii) mtSC enables cross-species single-cell assignment, especially for specific tissues with very few sequencing datasets available for a specific species. These two characteristics of mtSC are of great potential utility when much more sequencing data on different species have accumulated in the future.

Single-cell assignment is important and challenging with an increasing number of complex cell atlases becoming available (34,35). Although many existing single-cell assignment methods have been presented, issues exist. For example, for rare cell types (<10 cells), most of the single-cell assignment methods, including the current version of scLearn (13) and mtSC, are not satisfactory. In the current study, these cell types were excluded because they contain limited information and are unreliable for subsequent assignment. Therefore, efficient cell assignment and detection of rare cell types remain challenging (36,37).

## DATA AVAILABILITY

The 27 single cell assignment benchmark datasets were curated from four studies including three tissues: Peripheral blood mononuclear cells (PBMCs) (21,22), the brain (23,24) and the pancreas (25–28) (Supplementary Table S1). The four pancreas datasets (25–28) and one of brain datasets (24) was collected in previous work of scmap (11) (https://hemberg-lab.github.io/scRNA.seq.datasets), and the other three brain datasets and seven datasets in ‘PBMC-Ding’ (21) were curated from the following benchmark study (14) (https://doi.org/10.5281/zenodo.3357167). The 12 datasets in ‘PBMC-Mereu’ (22) were from GSE133549, and the corresponding RData file can be downloaded in https://www.dropbox.com/s/i8mwmyymchx8mn8/sce.all_classified.technologies.RData?dl=0. All these datasets were converted into Bioconductor SingleCellExperiment (http://bioconductor.org/packages/SingleCellExperiment) class objects. mtSC is developed as python package available at https://github.com/bm2-lab/mtSC, built in with 27 datasets within 3 tissues and pre-trained models, which can be utilized directly to facilitate a broad applications of single cell assignment with multiple references.

## Supplementary Material

gkab380_Supplemental_Files
