# Supplementary material for: Integrating multiple references for single-cell assignment
Source: Nucleic Acids Res. 2021 May 25;49(14):e80. doi: 10.1093/nar/gkab380 (PMC8373058; doi:10.1093/nar/gkab380)
Supplement: gkab380_Supplemental_Files [file gkab380_supplemental_files.zip › Supplementary materials.docx]

**Supplementary Materials for “Integrating multiple references for single cell assignment”.**

**The PDF files include:**

Supplementary Figures S1 to S4

Legends for Supplementary Tables S1 to S15


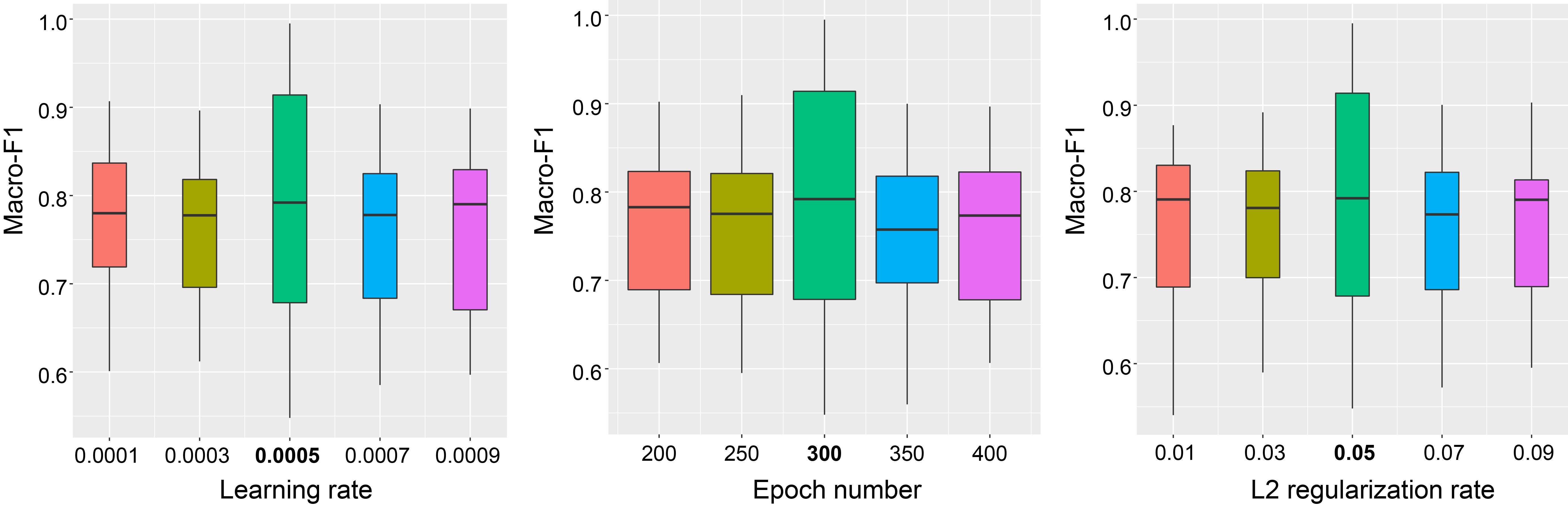


**Supplementary Figure S1.** The sensitive analysis on the parameters of mtSC, including learning rate, epoch number and l2 regularization rate. The bold values are the default values of parameters of mtSC.


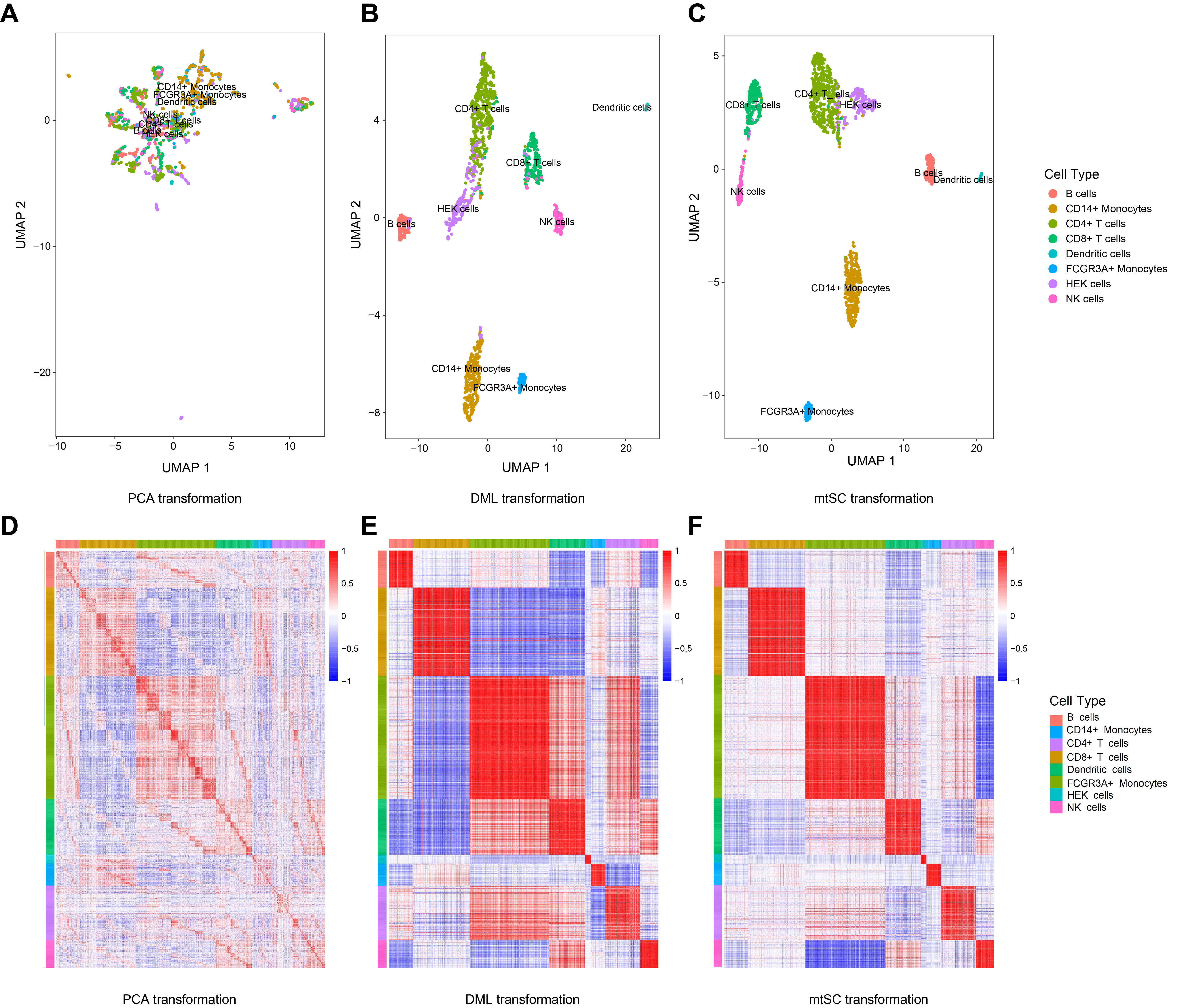


**Supplementary Figure S2.** Comparison of the clustering results among the transformations of PCA, DML and mtSC for dataset “Pbmc_mcSCRB-Seq”. **(A)** The visualization of clustering results with UMAP after PCA transformation for “Pbmc_mcSCRB-Seq” dataset. **(B)** The visualization of clustering results with UMAP after DML transformation for “Pbmc_mcSCRB-Seq” dataset. **(C)** The visualization of clustering results with UMAP after mtSC transformation for “Pbmc_mcSCRB-Seq” dataset. **(D)** Similarity heatmap calculated with the Pearson correlation coefficient after PCA transformation for “Pbmc_mcSCRB-Seq” dataset. **(E)** Similarity heatmap calculated with the Pearson correlation coefficient after DML transformation for “Pbmc_mcSCRB-Seq” dataset. **(F)** Similarity heatmap calculated with the Pearson correlation coefficient after mtSC transformation for “Pbmc_mcSCRB-Seq” dataset.


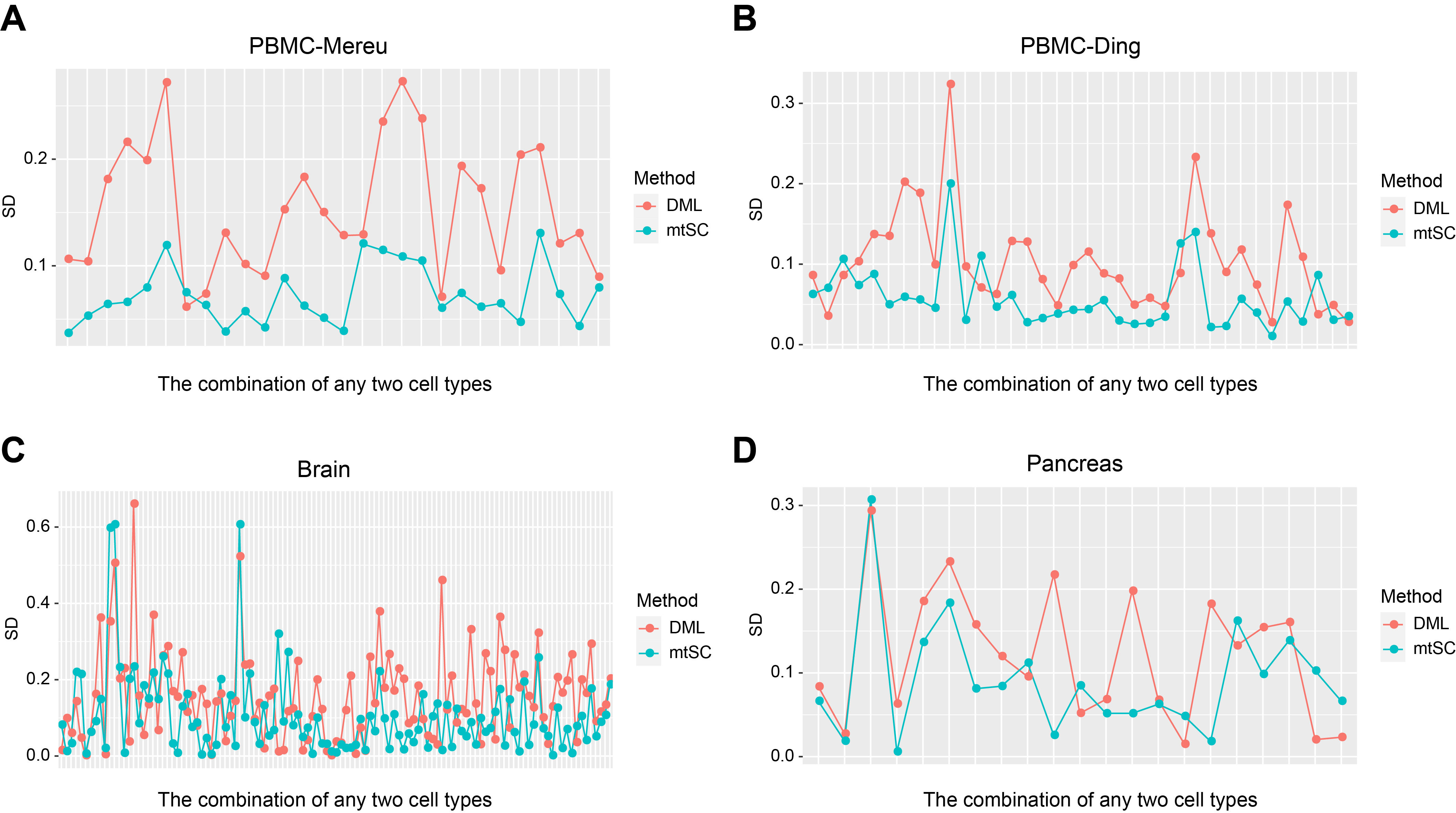


**Supplementary Figure S3.** The detailed information for the comparison of SD of similarity between any two cell types for the whole four studies. **(A)** The comparison of SD of similarity between any two cell types within “PBMC-Mereu” transformed by DML and mtSC. SD, standard deviation. **(B)** The comparison of SD of similarity between any two cell types within “PBMC-Ding” transformed by DML and mtSC. **(C)** The comparion of SD of similarity between any two cell types within “Brain” transformed by DML and mtSC. **(D)** The comparison of SD of similarity between any two cell types within “Pancreas” transformed by DML and mtSC.


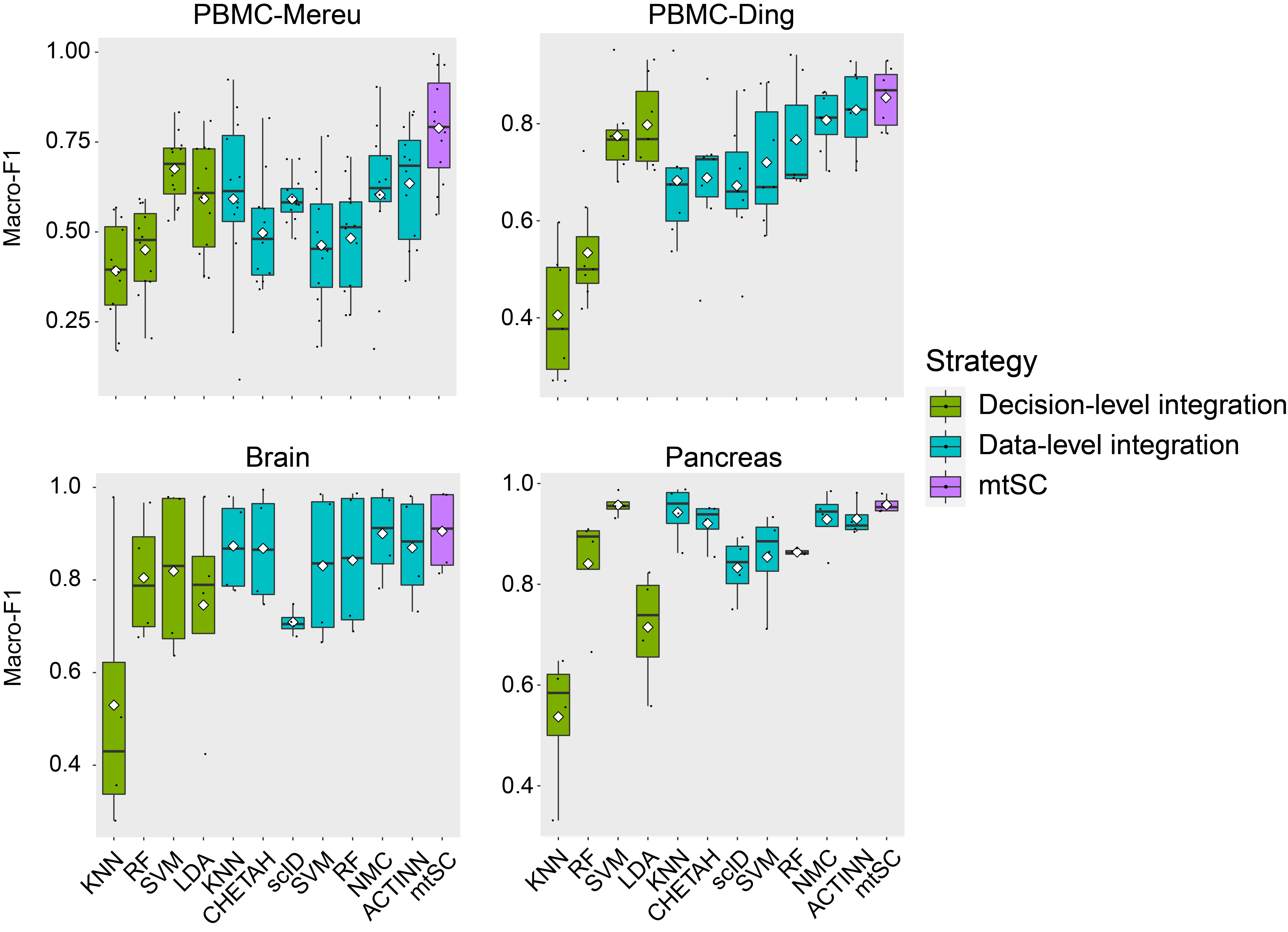


**Supplementary Figure S4.** The macro-F1 scores of other single reference based comparable tools with different integration strategies for “PBMC-Mereu”, “PBMC-Ding”, “Brain” and “Pancreas”, respectively. The white diamond represents the mean value.

**Legends for Supplementary Table S1 to S15:**

**Supplementary Table S1** The test datasets for different evaluations in multiple reference single cell assignment.

**Supplementary Table S2** The sensitive analysis on the parameters of mtSC, including learning rate, epoch number and l2 regularization rate. This supplementary table is corresponding to Supplementary Figure S1.

**Supplementary Table S3** The comparison of SD of similarity between any two cell types within “PBMC-Mereu” transformed by DML and mtSC. This supplementary table is corresponding to Figure 2G and Supplementary Figure S3A.

**Supplementary Table S4** The comparison of SD of similarity between any two cell types within “PBMC-Ding” transformed by DML and mtSC. This supplementary table is corresponding to Figure 2H and Supplementary Figure S3B.

**Supplementary Table S5** The comparison of SD of similarity between any two cell types within “Brain” transformed by DML and mtSC. This supplementary table is corresponding to Figure 2I and Supplementary Figure S3C.

**Supplementary Table S6** The comparison of SD of similarity between any two cell types within “Pancreas” transformed by DML and mtSC. This supplementary table is corresponding to Figure 2J and Supplementary Figure S3D.

**Supplementary Table S7** The macro-F1 scores of four strategies with “PBMC-Mereu”, “PBMC-Ding”, “Brain” and “Pancreas” separately. This supplementary table is corresponding to Figure 3A.

**Supplementary Table S8** The macro-F1 scores of all methods with different integration strategy in “PBMC-Mereu”, “PBMC-Ding”, “Brain” and “Pancreas” separately. This supplementary table is corresponding to Figure 3B.

**Supplementary Table S9** The macro-F1 scores of other single reference based methods with different integration strategy in “PBMC-Mereu”, “PBMC-Ding”, “Brain” and “Pancreas” separately. This supplementary table is corresponding to Supplementary Figure S4.

**Supplementary Table S10** The macro-F1 of each cell type for different methods on “PBMC-Mereu” and “PBMC-Ding” studies separately. This supplementary table is corresponding to Figure 3C.

**Supplementary Table S11** The macro-F1 of Seurat v3 and mtSC for cell types only appear in one of the multiple references for “Brain” datasets. This supplementary table is corresponding to Figure 3D.

**Supplementary Table S12** Single CPU (SingleR, scmap-cluster and Seurat v3) and single GPU (mtSC) consuming time to train references for existing tools. This supplementary table is corresponding to Figure 3E.

**Supplementary Table S13** Single CPU (SingleR, scmap-cluster, Seurat v3 and mtSC) consuming time to train references for existing tools. This supplementary table is corresponding to Figure 3F.

**Supplementary Table S14** The macro-F1 scores of mtSC as the number of reference datasets increases for “PBMC-Ding”. This supplementary table is corresponding to Figure 4.

**Supplementary Table S15** The macro-F1 scores of mtSC for cross-species single cell assignment. This supplementary table is corresponding to Figure 5.
