# Supplementary material for: Integrating multiple references for single-cell assignment
Source: Nucleic Acids Res. 2021 May 25;49(14):e80. doi: 10.1093/nar/gkab380 (PMC8373058; doi:10.1093/nar/gkab380)
Supplement: gkab380_Supplemental_Files [file gkab380_supplemental_files.zip › Supplementary Table S1.docx]

**Supplementary Table S1 The test datasets for different evaluations in multiple reference single cell assignment**

| Dataset | Description | No. of cells after QC | No. of cell populations | Protocol | Reference |
| --- | --- | --- | --- | --- | --- |
| Baron_human | Human pancreas | 8562 | 13 | inDrop | (25) |
| Muraro | Human pancreas | 2110 | 8 | CEL-Seq2 | (26) |
| Segerstolpe | Human pancreas | 2126 | 9 | SMART-Seq2 | (27) |
| Xin | Human pancreas | 1491 | 4 | SMARTer | (28) |
| Tasic | Mouse cortex | 695 | 4 | SMARTer | (24) |
| AMB | Primary mouse visual cortex | 4351 | 11 | SMART-Seq v4 | (23) |
| VISp | Mouse primary visual cortex | 12446 | 16 | SMART-Seq v4 | (23) |
| ALM | Mouse anterior lateral motor area | 7742 | 16 | SMART-Seq v4 | (23) |
| MTG | Human middle temporal gyrus | 13907 | 17 | SMART-Seq v4 | (23) |
| Pbmc_10Xv2 | PBMC | 9683 | 9 | 10X version 2 | (21) |
| Pbmc_10Xv3 | PBMC | 3188 | 8 | 10X version 3 | (21) |
| Pbmc_CL | PBMC | 526 | 7 | CEL-Seq2 | (21) |
| Pbmc_DR | PBMC | 6584 | 9 | Drop-Seq | (21) |
| Pbmc_iD | PBMC | 6287 | 7 | inDrop | (21) |
| Pbmc_SM2 | PBMC | 475 | 6 | SMART-Seq2 | (21) |
| Pbmc_SW | PBMC | 3706 | 7 | Seq-Well | (21) |
| Pbmc_C1HT-medium | PBMC | 1406 | 8 | C1HT-medium | (22) |
| Pbmc_C1HT-small | PBMC | 1330 | 8 | C1HT-small | (22) |
| Pbmc_CEL-Seq2 | PBMC | 834 | 7 | CEL-Seq2 | (22) |
| Pbmc_Chromium2 | PBMC | 1509 | 8 | Chromium2 | (22) |
| Pbmc_Chromium | PBMC | 1287 | 8 | Chromium | (22) |
| Pbmc_ddSEQ | PBMC | 1949 | 8 | ddSEQ | (22) |
| Pbmc_Drop-Seq | PBMC | 1922 | 8 | Drop-Seq | (22) |
| Pbmc_ICELL8 | PBMC | 1597 | 9 | ICELL8 | (22) |
| Pbmc_inDrop | PBMC | 606 | 7 | inDrop | (22) |
| Pbmc_MARS-Seq | PBMC | 1276 | 8 | MARS-Seq | (22) |
| Pbmc_mcSCRB-Seq | PBMC | 1592 | 8 | mcSCRB-Seq | (22) |
| Pbmc_Quartz-Seq2 | PBMC | 693 | 8 | Quartz-Seq2 | (22) |
